# Living alone and positive mental health: a systematic review

**DOI:** 10.1186/s13643-019-1057-x

**Published:** 2019-06-07

**Authors:** Nina Tamminen, Tarja Kettunen, Tuija Martelin, Jaakko Reinikainen, Pia Solin

**Affiliations:** 1 WHO Collaborating Centre for Mental Health Promotion, Prevention and Policy, Mental Health unit, National Institute for Health and Welfare, Helsinki, Finland; 2 Department of Health Sciences, University of Jyvaskyla, Jyväskylä, Finland; 3 Research Centre for Health Promotion, University of Jyvaskyla, Jyväskylä, Finland; 4 Central Finland Health Care District, Jyväskylä, Finland; 5 Equality and Inclusion unit, National Institute for Health and Welfare, Helsinki, Finland; 6 Public Health Evaluation and Projection unit, National Institute for Health and Welfare, Helsinki, Finland

**Keywords:** Living alone, Positive mental health, Systematic literature review

## Abstract

**Background:**

Living alone has become more common in today’s societies. Despite the high number of the population living alone, research directed towards the mental wellbeing issues related to living alone has been limited. This systematic literature review aimed to assess the association between living alone and positive mental health.

**Methods:**

We conducted searches in Medline, Web of Science, Cochrane Library, CINAHL, PsycINFO, and other complementary databases from January 1998 to May 2019. Randomised trials and observational studies investigating adults over 18 years of age and living alone (defined as living in a single household or a household size of one person) were eligible. The primary outcome was positive mental health, defined as comprising both hedonic and eudaimonic elements of mental wellbeing, and it was measured with the Warwick-Edinburgh Mental Well-being Scale and/or theWHO-5 Index. Two reviewers independently screened and selected data; one reviewer extracted data, and the second checked the extracted data. A narrative synthesis described the quality and content of the evidence. Included studies were appraised using relevant Joanna Briggs Institute checklist.

**Results:**

A total of 4 cross-sectional studies (22,591 adult participants) were included after screening of 341 titles and abstracts and 46 full-text articles. These studies were conducted in Europe and were published between 2014 and 2017. The studies differed in their measurements of positive mental health (WHO-5 Well-Being Index, 3 studies; WEMWBS, 1 study), sources of data (1 regional, 1 national, and 2 European-level studies), and study populations (regional study, adults over 65 years of age; national-level study, mental health nurses over 21 years of age; European-level studies, employees between 15 and 65 years of age and adults over 18 years of age). A potential association between living alone and low positive mental health was found in three out of the four studies. Our findings were limited as the number of included studies was low and the quality of evidence varied across studies.

**Conclusions:**

This review allows a limited look at the association between living alone and positive mental health. Because the number of included studies was low and the quality of evidence varied across studies, further research is warranted.

**Electronic supplementary material:**

The online version of this article (10.1186/s13643-019-1057-x) contains supplementary material, which is available to authorized users.

## Background

Living alone has become more common in today’s societies. In 2017, one third (33.6%) of households in the EU (European Union) and around 40% of households in the Nordic countries (with the exception of Iceland) were single-person households [1]. The number of people living alone is likely to continue to increase globally among both older people and working adults [2].

The definitions of living alone or being single may vary. Nowadays, official marital status no longer necessary reflects an individual’s living arrangements as single, divorced, and widowed persons may live alone or with other people such as a partner, children, parents, or other unrelated persons. Thus, more than official marital status, living arrangements may better describe one’s social bonds. In addition, people living alone do not constitute a uniform group. People living alone may be at very different life stages depending on their age, gender, education, and work status. Moreover, living arrangements can change several times during an individual’s life course. In this review, living alone is understood as only one person living in a household at the time of the research, in other words, a household size of one person. As Jamieson et al. stated [2] ‘The essence of living alone is simple: nobody else lives in the same living space or routinely shares everyday domestic life’ (p. 5).

Earlier studies have produced conflicting results concerning the association between living alone and mental health. According to some studies, living alone does not constitute a risk factor to mental health [3, 4]. On the other hand, some authors have reported associations with depression, poorer experienced health and quality of life, and experiences of loneliness [5–8]. Further, research shows that people living alone face challenges that may place a potential burden on their mental wellbeing, such as financial difficulties and higher living costs as they do not have the scale advantage of those living with another adult [5, 9]. There is therefore a need to further examine the relationship between living alone and positive mental health.

The term *positive mental health* is often used and understood in policy and academic literature as interchangeable with the term *mental wellbeing* [10, 11]. Furthermore, in research, both of these concepts have sometimes been operationalised under the concept of *subjective wellbeing* [12–14]. In this review, positive mental health is understood as being interchangeable with mental wellbeing or subjective wellbeing.

Positive mental health is based on the assumption that mental health is something positive, consists of wellbeing, and is more than the absence of mental illness [15]. It is recognised as a key resource for health and wellbeing [16]. Positive mental health has been shown to be associated with mortality, physical health, social functioning, and academic achievement, as well as with mental illness [13, 17, 18]. It is currently receiving increased attention in research, policymaking, and clinical practice [19], and it has been recognised as a priority research area in public mental health [20]. Positive mental health is conceived as a multi-faceted construct that comprises both hedonic and eudaimonic elements. The hedonic perspective focuses on subjective experience of happiness and life satisfaction. The eudaimonic perspective, on the other hand, views wellbeing as something more than subjective feelings, and focuses on psychological functioning and self-realisation [11, 12]. Positive mental health includes individual resources, such as self-esteem, optimism and a sense of mastery and coherence; the ability to initiate, develop and sustain mutually satisfying personal relationships; and the ability to cope with adversities [21].

Efforts to investigate positive mental health have been hampered by a shortage of valid instruments suitable for measuring the attributes of positive mental health. The Warwick-Edinburgh Mental Well-being Scale (WEMWBS) measures positive mental health, covering both the hedonic and eudaimonic aspects of mental wellbeing. The scale consists of 14 positively worded items covering ‘positive affect (feelings of optimism, cheerfulness, relaxation), satisfying interpersonal relationships, and positive functioning (energy, clear thinking, self-acceptance, personal development, competence and autonomy)’ ([22], p. 3). The scale was developed to enable the monitoring of mental wellbeing in the general population and the evaluation of projects, programmes, and policies which aim to improve mental wellbeing. There is also a shortened version of the WEMWBS with seven items (SWEMWBS) [23]. The scale has been used in national surveys in Scotland and England [24, 25]. In the Scottish Health Survey, in the 2012 and 2013 combined dataset [24], the WEMWBS scores were lowest for adults who were separated. In the Health Survey for England, in the 2010 and 2011 combined dataset [25], people who were single, divorced, or widowed had lower wellbeing scores than those who were married or lived as a couple. Both studies described marital status and did not differentiate those who were living alone for real.

An instrument similar to the WEMWBS is the WHO-5 Well-Being Index [26]. The index shares common features with the WEMWBS measurement, capturing positive affect and wellbeing [22, 27] and measuring both the hedonic and eudaimonic aspects of wellbeing [26]. The index is a positively worded 5-item questionnaire measuring current mental wellbeing. The scale was first presented at a WHO (World Health Organization) meeting in Stockholm in 1998. Since then, the WHO-5 Well-Being Index has been validated in a number of studies with regard to both its clinical and psychometric validity [28].

Despite the high number of the population living alone, research directed specifically towards mental wellbeing issues related to living alone has been limited. The objective of this review is to collect and assess the body of empirical research on the association between living alone and positive mental health. The review concentrates on adults living alone and on two indicators that measure positive mental health, the WEMWBS and the WHO-5 Index as they both comprise the hedonic and eudaimonic aspects of mental wellbeing.

## Methods

This systematic review was reported in accordance with the reporting guidance provided in the Preferred Reporting Items for Systematic Review and Meta-Analysis (PRISMA) statement [29] (see the checklist in Additional file 1). The review protocol is included as Additional file 2.

### Eligibility criteria

Studies were eligible for inclusion if they reported original research (such as randomised controlled trials, observational studies, or mixed methods studies) and the study population included adults (those over 18 years of age) living alone. *Living alone* could be covered by belonging to the category of ‘living alone’, ‘living in a single household’, or ‘a household size of one person’. Studies considering positive mental health as an outcome and/or including the WEMWBS/SWEMWBS and/or the WHO-5 positive mental health measurement scales were included. Studies conducted from 1998 onwards (the WHO-5 measurement was introduced in 1998) were eligible. Only fully published, peer-reviewed papers reported in English were included.

### Information sources and the literature search

The literature search was performed by an information specialist in October and November 2017. Sixteen databases were searched from 1998 to November 2017 to identify English language publications. The main electronic databases included: Medline, Web of Science, Cochrane Library, the Cochrane Database of Systematic Reviews, Cumulative Index to Nursing and Allied Health Literature (CINAHL), and PsycINFO. Complementary databases included ASSIA (Applied Social Sciences Index and Abstracts), the International Bibliography of the Social Sciences (IBSS), the Political Science Database, the Social Science Database, the Sociology Database, the Education Database, Sociological Abstracts and Social Services Abstracts, Academic Search Elite, SocINDEX, AgeLine and Urban Studies Abstracts, and one search engine, Google Scholar. The search was updated in May 2019 regarding the main 5 databases: Medline, Web of Science, Cochrane Library, CINAHL and PsycINFO. The search strategy was developed with the team’s professional health science librarian and search algorithms were tailored for each database (see the search strategies by database in Additional file 3). Searches were piloted, and as a result, broader descriptions of living alone and positive mental health were used to ensure as wide as possible coverage in the review. The final strategy consisted of two search aspects: (1) search terms related to living alone: *living alone*, *single*-*living*, *one*-*person households*, *singlehood*, *single people*, *single persons*, *single men*, *single women* and (2) search terms related to positive mental health: *positive mental health*, *mental wellbeing*, *subjective wellbeing*, *Warwick*-*Edinburgh Mental Well*-*being Scale*, *WHO*-*5 Well*-*being Index*.

### The screening and selection procedure

Two researchers (NT, PS) independently carried out the screening process. Any discrepancies were discussed until there was a consensus. The screening took place in two steps. In Step 1, all titles and abstracts were screened for relevance and eligibility. Articles that were not relevant or did not meet the inclusion criteria were removed. Articles that had insufficient information in the title and the abstract to determine their relevance were screened in Step 2. In Step 2, the full texts of the remaining articles were reviewed for relevance and in reference to the inclusion criteria.

### Data collection

A data extraction form was developed to enable the collection of data. One review author extracted the data (with the assistance of the Atlas.ti data analysis software) and the second author checked the extracted data. The following information was extracted from each study: (1) study identification features: authors, title, country, year; (2) study characteristics: aims/objectives, study design, data source, data collection method; (3) population characteristics: age, gender, sample size; (4) outcome results: measured positive mental health, scales used, key findings; and (5) study limitations/strengths.

### Quality assessment

To assess the risk of bias in individual studies, a methodological quality critical appraisal checklist proposed by the Joanna Briggs Institute (JBI) systematic review methods manual [30, 31] was used. This tool for observational studies reporting prevalence data considers the following: sample frame appropriateness, recruitment appropriateness, sample size, descriptions of subjects and setting, coverage of data analysis, ascertainment and measurement of the condition, the thoroughness of reporting statistical analysis, and the adequacy and management of the response rate (see Additional file 4). We judged each individual domain as having a high, low, or unclear risk of bias. Two reviewers (NT, PS) independently assessed the studies. Discrepancies were discussed and resolved through finding consensus. The results of the appraisal were used to inform the synthesis and interpretation of the review results.

### Data analysis

The data from each study (e.g. the study characteristics, context, participants, outcomes and findings) were used to build evidence tables for an overall description of the included studies. As study populations and data sources differed between the included studies, a quantitative analysis was considered inappropriate and a narrative synthesis was conducted instead.

## Results

**Fig. 1:**
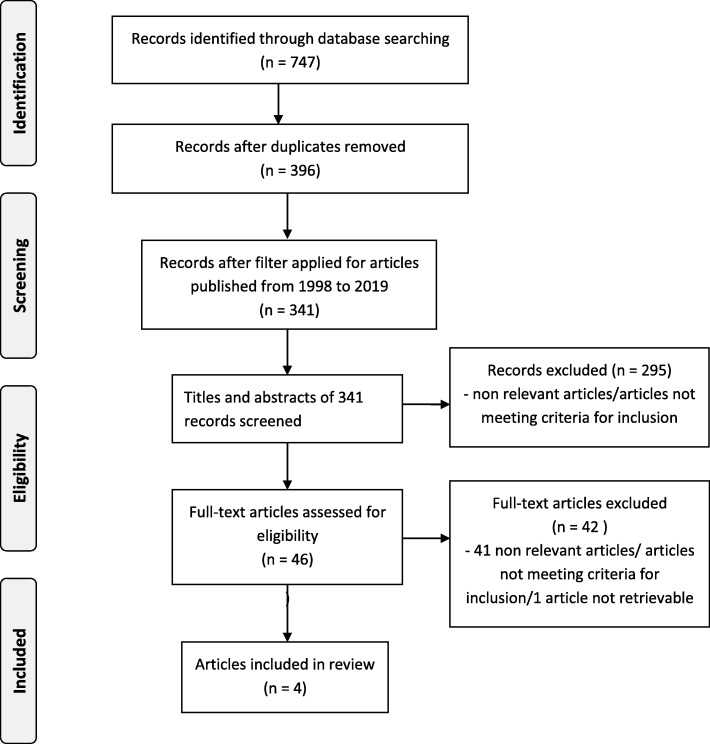
The PRISMA flowchart of the study selection process

The literature search identified 341 records, of which the full texts of 46 were examined and 42 of these were then excluded. Accordingly, we included four studies. A PRISMA flowchart documenting the process of study selection is shown in Fig. 1.

### The characteristics of the studies

Two of the studies were European-level studies [32, 33]: one was carried out in Southern Germany [34] and one in the UK [35]. All the studies were published within the last few years (between 2014 and 2017) (Table 1).

**Table 1:** The characteristics of the included studies

Author, year	Country	Study design	Data source; type of tool	Study population; sample size	Positive mental health measure	Key findings
De Moortel et al., 2015 [32]	European	Cross-sectional	European Social Survey (ESS); face-to-face interviews.	Male (*n* = 7119) and female (*n* = 6988) employees, aged 15–65 years; approx. 12% described as living alone (percentage stated per welfare regime).	WHO-5 Well-Being Index: three items	Good mental wellbeing (positive mental health) was less prevalent for women living alone, compared to women without children living with a partner who did half or more of the household labour (prevalence ratio among women: 0.81 (95% confidence interval 0.72–0.90) and among men: 0.98 (0.89–1.08)).
Dreger et al.,2014 [33]	European	Cross-sectional	European Quality of Life Survey (EQLS); face-to-face interviews.	Men (*n* = 21,066) and women (*n* = 22,569), aged 18 years and over; 8926 men and 10,749 women described as living alone.	WHO-5 Well-Being Index	Living alone was associated with positive mental health in both genders. Living without a partner was significantly associated with low positive mental health among both genders (odds ratio among men: 1.18 (95% confidence interval 1.07–1.30) and among women: 1.17 (1.09–1.25)).
Lukaschek et al., 2017 [34]	Southern Germany	Cross-sectional	The KORA (Cooperative Health Research in the Region of Augsburg) -Age study; telephone interview or postal questionnaire.	Participants aged 65 years or older: *n* = 3602 (men *n* = 1750; women *n* = 1822); 335 men and 852 women described as living alone.	WHO-5 Well-Being Index	The impact of living alone on low subjective wellbeing (positive mental health) was significant only in women. Living alone increased the odds of having low subjective wellbeing in women (odds ratio: 1.43 (95% confidence interval 1.10–1.87)), but not significantly in men (1.19 (0.85–1.68)).
Oates et al., 2017 [35]	UK	Cross-sectional	UK mental health nurses (MHN); online questionnaire.	Female (*n* = 159) and male (*n* = 65) mental health nurses; living alone (*n* = 37, including both sexes).	Warwick-Edinburgh Mental Well-Being Scale (WEMWBS)	Household size was not significantly correlated with subjective wellbeing (positive mental health), although those living alone had lower mean subjective wellbeing measure score. Mean score of those living alone: 46.69 (standard deviation 8.30), living with 1 person: 48.88 (7.95), living with 2–3 others: 46.89 (8.54) and living with 4+ others: 47.60 (8.33).

The included studies were all cross-sectional in design. One study used the European Social Survey (ESS) as the data source for their study [32], one used the dataset from the European Quality of Life Survey (EQLS) [33], one from the KORA-Age study (KORA stands for Cooperative Health Research in the Region of Augsburg) [34], and one study carried out their own survey [35]. The survey tools varied encompassing face-to-face interviews [32, 33], an online questionnaire [35], and a telephone interview and a postal questionnaire [34].

#### Study populations

Three of the studies included wide study populations which described the number or percentage of those living alone (see Table 1 for study population sizes). De Moortel et al. [32] studied male and female employees between 15 and 65 years of age; the study population of Dreger et al. [33] consisted of men and women 18 years of age and older; and Lukaschek et al. [34] investigated a population that included men and women 65 years of age or older. The study of Oates et al. [35] involved female and male mental health nurses over 21 years of age. Only a small number of the participants lived alone.

#### Positive mental health measures and study outcomes

Positive mental health was measured with the WHO-5 Well-Being Index in three of the studies [32–34] and with the WEMWBS in one study [35].

Regarding study outcomes, three of the studies reported associations between living alone and positive mental health. Dreger et al. [33] found that living without a partner was significantly associated with poor positive mental health for both genders in a model adjusted for sociodemographic and psychosocial factors and in a model adjusted for sociodemographic, psychosocial, and material factors. They used a large dataset provided by the EQLS, producing a large study population of those living alone, thus providing strength to their study results. This study was the only study that found associations in both women and men.

De Moortel et al. [32] found that good mental wellbeing (positive mental health) was less prevalent for women living alone, compared to women without children living with a partner who did half or more of the household labour (state corporatist/family support welfare regimes). The study employed a large dataset provided by the ESS. The ESS dataset only contained three items of the WHO-5 Well-Being Index to measure mental wellbeing (i.e. positive mental health). The researchers of the study, however, were confident of its internal consistency and the use of the three-item scale to measure mental wellbeing. Lukaschek et al. [34] reported similar findings regarding women living alone. They found that the impact of living alone on low subjective wellbeing (positive mental health) was only significant in women. Living alone increased the odds of having low subjective wellbeing in women but not in men. The study population in their research was again different from the other included studies; the study participants were older men and women between 65 and 90 years of age. The study population size was fairly large in their study. As a result of their findings, they suggested that living alone may have a negative effect on the wellbeing of older women. The researchers proposed that women place greater value on social ties than men, signifying that living alone could make older women vulnerable to lower subjective wellbeing.

Oates et al. [35] found no significant correlations between living alone and positive mental health. They reported that household size was not significantly correlated with subjective wellbeing (positive mental health). Their study was the only study to use the WEMWBS measurement to assess positive mental health. The sample size in their study was fairly small, and the study concerned a very specific study population: mental health nurses in the UK.

### The quality of the included studies

We assessed the risk of bias in the included studies in nine domains. The results of the critical appraisal are presented in Table 2. All the included studies had their target population appropriately framed; however, two of them (those by De Moortel et al. and Dreger et al.) did not provide detailed information regarding sample recruitment and were thus considered to have an unclear risk of bias in this domain. One study (by Oates et al.) was assigned a high risk of bias regarding the precision of the results as the sample size was small. Two of the studies had a high risk of coverage bias as the response rates were either low (in the study by Oates et al.) or varied between subgroups—some having a higher response rate and some having a lower response rate (in the study by Dreger et al.). In terms of factors that reduced the risk of bias, all the included studies employed appropriate statistical tests reporting the analyses made. One study (that of De Moortel et al.) was, nonetheless, considered to have a high risk of measurement bias as they used only a part of a validated measurement.

**Table 2:** The critical appraisal results of the included studies using the JBI-Prevalence Critical Appraisal Checklist

Study	Was the sample frame appropriate to address the target population?	Were study participants sampled in an appropriate way?	Was the sample size adequate?	Were the study subjects and the setting described in detail?	Was the data analysis conducted with sufficient coverage of the identified sample?	Were valid methods used for the identification of the condition?	Was the condition measured in a standard, reliable way for all participants?	Was there appropriate statistical analysis?	Was the response rate adequate, and if not, was the low response rate managed appropriately?
De Moortel et al., 2015 [32]	Yes	Unclear	Yes	Yes	Yes	No	Yes	Yes	Unclear
Dreger et al., 2014 [33]	Yes	Unclear	Yes	Yes	No	Yes	Yes	Yes	Yes
Lukaschek et al., 2017 [34]	Yes	Yes	Yes	Yes	Yes	Yes	Yes	Yes	Yes
Oates et al., 2017 [35]	Yes	Yes	No	Yes	No	Yes	Yes	Yes	No

## Discussion

This review aimed to collect and assess empirical data on the association between living alone and positive mental health, and to highlight possible shortages in this field of research. Despite including an extensive number of databases in the review and a comprehensive search strategy, the search resulted in a surprisingly low number of studies (four) that focused on positive mental health and living alone, thus indicating a shortage of research investigating the association. Positive mental health as such is a relatively new concept and research area, and according to this review, studies concentrating specifically on the positive mental health of those living alone seem to be very scarce. This novelty of the research area was supported by the finding that all the included studies were published within the last few years.

As the number of included studies was low and the quality of evidence varied across studies, the review only allows a narrow look at the associations of living alone and positive mental health. Three of the studies reported associations between living alone and low positive mental health [32–34]. These studies had large or fairly large population sample sizes. The study that found no correlation had, on the other hand, a low response rate with a small sample size [35], thus contributing to a high risk of bias regarding the precision of the results. This may suggest that in order to find potential associations, the study sample needs to be based on adequate response rates and be of a fairly large size.

Some gender differences were found in the study findings: two of the studies found associations in women but not in men [32, 34]. The national surveys of Scotland and England [24, 25], as well as the recently conducted National FinHealth 2017 Study [36], however, did not find differences in positive mental health scores between women and men. It is worth noting that none of these studies distinctly classified those living alone (i.e. a household size of one person). Interestingly, research on mental illness has found that living arrangements are strongly associated with mental health and particularly among men [5]: compared with married persons, persons living alone had higher odds of psychological distress and psychiatric disorders. These puzzling results may suggest that the correlates of positive mental health may be different from the correlates of mental illness [11], calling for further investigations into positive mental health outcomes in general, as well as into the positive mental health status of people living alone.

Given the range of the eligibility criteria, the studies differed in their measurements of positive mental health. Two measurements of positive mental health were used in the included studies: the WEMWBS and the WHO-5 Well-Being Index. Even though the two instruments share the same characteristics—both are positively worded and both measure the hedonic and eudaimonic aspects of mental wellbeing—caution needs to be taken when comparing study results between two ultimately different measurements [37]. In addition to this, a partial measurement was employed in one study, thus producing a high risk of measurement bias. These notions add to the weak evidence found in the review regarding associations between living alone and positive mental health. To conclude, no general conclusions can be made from the included studies and their study results; they must be evaluated individually and within their study context.

### The limitations of the review

This review has a number of limitations affecting its validity. Firstly, due to the resources available, the systematic search only focused on articles published in English, possibly leaving unidentified studies published in other languages outside the review. In a similar way, grey literature and unpublished articles were not systematically searched for. This could contribute to publication bias. To minimise the effect of this limitation and to ensure as wide as possible coverage in the review, a high number of databases were searched and broader descriptions of the key terms were used. Secondly, as all the studies included in the review were cross-sectional in study design, it is impossible to make conclusions with regard to causality. In addition, the study populations were diverse and two different measures were used to assess positive mental health, and this thus affected the applicability of this review. However, these types of studies can provide evidence of the health status of a specified population group in a certain location at a given time [38]. Thirdly, all the included studies involved participants self-reporting, either by answering a questionnaire or being interviewed, which can lead to information bias. Consequently, care must be taken in interpreting such information as there is a tendency for respondents to provide what they believe to be socially acceptable answers, especially with regard to health conditions associated with taboos [38].

## Conclusions

The review findings permitted a limited look at the association between living alone and positive mental health. A potential association with living alone and low positive mental health was perceived in those studies where the sample size was large or fairly large. It is therefore clear that more research is needed in study samples of appropriate sizes. As the number of people living alone is likely to continue to increase, it is recommended to investigate the issue on a much greater scale. An example would be to study the associations of living alone and positive mental health in large population studies such as the National FinHealth 2017 Study [36] carried out in Finland.

Positive mental health has been recognised as a key resource for health and wellbeing, and it may have a beneficial influence not just on health and quality of life but also on social functioning and productivity. New knowledge produced by vigorous research can be of use in policy development and decision-making in relation to those living alone and their health and wellbeing. As more people both in Europe and globally are living alone, the issue is of high societal importance.

## Additional files

Additional file 1: PRISMA checklist. This file presents the PRISMA 2009 checklist employed in the study. (DOC 65 kb)

Additional file 2: Review protocol. This file presents the protocol of the study. (DOC 93 kb)

Additional file 3: Search strategies by database. This file presents the search strategies by each database employed in the study. (DOCX 35 kb)

Additional file 4: JBI critical appraisal checklist for prevalence studies. This file presents the JBI critical appraisal checklist for studies reporting prevalence data. (DOCX 17 kb)

## Data Availability

Data sharing is not applicable to this article as no datasets were generated or analysed during the current study.

## Abbreviations

ASSIA: Applied Social Sciences Index and Abstracts
CINAHL: Cumulative Index to Nursing and Allied Health Literature
EQLS: European Quality of Life Survey
ESS: European Social Survey
EU: European Union
IBSS: International Bibliography of the Social Sciences
JBI: Joanna Briggs Institute
KORA: Cooperative Health Research in the Region of Augsburg
PRISMA: Preferred Reporting Items for Systematic Review and Meta-Analysis
SWEMWBS: Short Warwick-Edinburgh Mental Well-being Scale
UK: United Kingdom
WEMWBS: Warwick-Edinburgh Mental Well-being Scale
WHO: World Health Organization
