# Supplementary material for: Living alone and positive mental health: a systematic review
Source: Syst Rev. 2019 Jun 7;8:134. doi: 10.1186/s13643-019-1057-x (PMC6555743; doi:10.1186/s13643-019-1057-x)
Supplement: Supplementary file 2 — Review protocol. This file presents the protocol of the study. (DOC 93 kb) [file 13643_2019_1057_MOESM2_ESM.doc]

**This checklist has been adapted from Table 3 in Moher D et al: Preferred reporting items for systematic review and meta-analysis protocols (PRISMA-P) 2015 statement. Systematic Reviews 2015 4:1**

Date: September 2017 (updated December 2017, March 2019)

| Section and topic | Item No | Checklist item |
| --- | --- | --- |
| ADMINISTRATIVE INFORMATION | | |
| Title: |  |  |
| Identification | 1a | Living alone and positive mental health: a systematic review |
| Registration | 2 | Non-registered. Protocol attached to the manuscript. |
| Authors: |  |  |
| Contact | 3a | Corresponding author: Nina Tamminen, nina.tamminen@thl.fi, WHO Collaborating Centre for Mental Health Promotion, Prevention and Policy, Mental Health unit, National Institute for Health and Welfare, Helsinki, Finland; University of Jyvaskyla, Department of Health Sciences, Finland.  Address: National Institute for Health and Welfare, P.O. Box 30, 00271 Helsinki, Finland  Pia Solin, pia.solin@thl.fi, WHO Collaborating Centre for Mental Health Promotion, Prevention and Policy, Mental Health unit, National Institute for Health and Welfare, Helsinki, Finland  Tuija Martelin, tuija.martelin@thl.fi, Equality and Inclusion unit, National Institute for Health and Welfare, Helsinki, Finland  Jaakko Reinikainen, jaakko.reinikainen@thl.fi, Public Health Evaluation and Projection unit, National Institute for Health and Welfare, Helsinki, Finland  Tarja Kettunen, tarja.h.kettunen@jyu.fi, University of Jyvaskyla, Research Center for Health Promotion, Finland; Central Finland Health Care District, Jyväskylä, Finland |
| Contributions | 3b | NT is the guarantor. All authors contribute to the conceptualisation of the review, development of the selection criteria, the search strategy and data extraction criteria. NT and PS carry out the quality assessment. NT drafts the manuscript; all authors also contribute to the drafting of the manuscript. All authors read, provide feedback and approve the final manuscript. |
| Support: |  |  |
| Sources | 5a | The systematic review funded by the Research Council for Health, Academy of Finland (grant number 308823); Information specialist from the National Institute for Health and Welfare, Finland, for technical support with the database search. |
| INTRODUCTION | | |
| Rationale | 6 | *“Despite its prevalence, living alone is one of the least discussed and, consequently, most poorly understood issues of our time.”* (Klinenberg 2012).  Living alone has become more common in today’s societies. In 2017, one third (33.6 %) of households in the EU and around 40% of households in the Nordic Countries (with the exception of Iceland) were single person households. The number of people living alone is likely to continue to increase globally among both older people and working adults.  In spite of the high number of population living alone, research on issues related to living alone has been limited focusing especially on older people and their well-being. More importantly, much of research has been family-oriented. However, people living alone face challenges such as lower income and higher living costs as they do not have the scale advantage as those living with another adult. In addition, people living alone experience more shortages in their well-being compared to those living with someone. Living alone is associated with various psychological and social challenges such as poorer experienced health, more psychological problems such as depression, poorer quality of life, experiences of loneliness and higher unemployment rates. However, research on mental well-being issues and positive mental health related to living alone, has been scarce.  Positive mental health bases on the assumption that mental health is something positive involving well-being and is not merely the absence of illness. Positive mental health is recognized as a key resource for well-being and is currently receiving increased attention in research, policy making, and clinical practice. Positive mental health is conceived as a multi-faceted construct that comprises both hedonic and eudaimonic elements. The hedonic perspective focuses on the subjective experience of happiness and life satisfaction. The eudaimonic perspective, on the other hand, views wellbeing as more than subjective feelings, and focuses on psychological functioning and self-realisation. Positive mental health includes individual resources, such as self-esteem, optimism and a sense of mastery and coherence; ability to initiate, develop and sustain mutually satisfying personal relationships; and ability to cope with adversities. Positive mental health has been shown to be associated with reduced mortality, better physical health and better academic achievement.  In order to fill the gap in research and knowledge on the issue, a systematic review is conducted on the associations between people living alone and positive mental health. Living alone is understood as only one person living in a household at the time of the research, i.e. a household size of one person. Studies considering positive mental health and including the WEMWBS and/or the WHO-5 positive mental health measurement scales are included. |
| Objectives | 7 | The aim is to collect and summarise the body of empirical research on the association of living alone and positive mental health. Target population: one-person households; condition: experienced positive mental health; context: studies conducted since 1998 (WHO-5 introduced) and using either WHO-5 or WEMWBS/SWEMWBS for measuring positive mental health. |
| METHODS | | |
| Eligibility criteria | 8 | Study design: any study design investigating the association between living alone and positive mental health: i.e. observational studies such as cohort and cross-sectional studies; intervention studies such as RCTs; quantitative, qualitative and mixed-methods designs are included.  Population: studies examining people (adults over 18 years of age) living alone: one-person households  Condition: experienced positive mental health / mental wellbeing understood as equivalent to positive mental health; measured with WHO-5 and/or WEMWBS/SWEMWBS (both comprises hedonic and eudaimonic elements).  Context: no geographical restrictions (see: language requirements).  Time restriction: studies conducted from 1998 onwards (WHO-5 measurement was introduced at the time).  Language: articles reporting in English only included.  Publications: only published, peer-reviewed papers reporting original research included, grey literature excluded. |
| Information sources | 9 | A search strategy is developed with the assistance of the team’s health science librarian, including planning the electronic databases to be employed. A pilot search is conducted to further develop the search strategy.  Several electronic databases are to be employed, including Medline, Web of Science, ASSIA (Applied Social Sciences Index and Abstracts), International Bibliography of the Social Sciences (IBSS), Political Science Database, Social Science Database, Sociology Database, Education database, Sociological Abstracts and Social Services Abstracts, Cochrane Library and Cochrane Database of Systematic Reviews, CINAHL, Academic Search Elite, SocINDEX, AgeLine, PsycINFO, Urban Studies Abstracts. (List updated Dec 2017 to include all employed databases, no omissions made to the list).  Also Google Scholar (search engine) to be employed.  Grey literature excluded. |
| Search strategy | 10 | A pilot search is conducted to further develop the search strategy and the inclusion criteria together with the research team. The specific search strategies for each database are created by the team’s health sciences librarian who has expertise in systematic review searching.  Both qualitative and quantitative studies will be sought. No study design, date or language limits are imposed on the initial search. Further filter is applied regarding the date: studies conducted from 1998 onwards included. Also, only studies in English are included due to resource limits.  Pilot search conducted 14.9.2017   | Living alone | AND | Positive mental health | AND | (Age) | | --- | --- | --- | --- | --- | | Living alone  live alone  Living single  single-living  one person household  single household  single people  single person |  | Positive mental health  Mental well-being  WEMWBS  SWEMWBS  "Warwick-Edinburgh Mental Well-being Scale" |  | Adult  age-group 18-  young adult  middle aged  working-aged  aged  old people  elderly |   **Web of Science 14.9.2017**  (TS=("Living alone") OR TS=(living NEAR/3 single*) OR TS=("one person" NEAR/2 household*) OR TS=(single NEAR/2 (household* OR people OR person*))) AND TS=("Positive mental health" OR "Mental wellbeing" OR "Mental well-being" OR WEMWBS OR SWEMWBS OR "Warwick-Edinburgh Mental Well-being Scale")  Indexes=SCI-EXPANDED, SSCI, A&HCI Timespan=1975-2017 Indexes=SCI-EXPANDED, SSCI, A&HCI Timespan=1975-2017Timespan: 1975-2017. Indexes: SCI-EXPANDED, SSCI, A&HCI.  Results 5, of which 1 adolescent-article  **Family & Society Studies Worldwide (EbscoHost) 14.9.2017**  # Query Limiters/Expanders Last Run Via Results  S1 TI living alone OR AB living alone OR SU living alone OR KW living alone  1,368  S2 TI ( one person household* OR one-person household* ) OR AB ( one person household* OR one-person household* ) OR SU ( one person household* OR one-person household* ) OR KW ( one person household* OR one-person household* )  257  S3 TI single* household* OR AB single* household* OR SU single* household* OR KW single* household*  1,201  S4 TI ( singlehood or "the single" ) OR AB ( singlehood or "the single" ) OR SU ( singlehood or "the single" ) OR KW ( singlehood or "the single" )  41,147  S5 S1 OR S2 OR S3 OR S4  42,532  S6 TI positive N2 (mental health) OR AB positive N2 (mental health) OR SU positive N2 (mental health) OR KW positive N2 (mental health)  341  S7 TI ( "Mental well-being" or "mental wellbeing" or "mental well being" ) OR AB ( "Mental well-being" or "mental wellbeing" or "mental well being" ) OR SU ( "Mental well-being" or "mental wellbeing" or "mental well being" ) OR KW ( "Mental well-being" or "mental wellbeing" or "mental well being" )  533  S8 TI ( "Psychologicall well-being" or "psychological wellbeing" or "psychological well being" ) OR AB ( "Psychologicall well-being" or "psychological wellbeing" or "psychological well being" ) OR SU ( "Psychologicall well-being" or "psychological wellbeing" or "psychological well being" ) OR KW ( "Psychologicall well-being" or "psychological wellbeing" or "psychological well being" ) Search modes - Boolean/Phrase Interface - EBSCOhost Research Databases  3,548  S9 TI ( WEMWBS OR SWEMWBS OR "Warwick-Edinburgh Mental Well-being Scale" ) OR AB ( WEMWBS OR SWEMWBS OR "Warwick-Edinburgh Mental Well-being Scale" ) OR SU ( WEMWBS OR SWEMWBS OR "Warwick-Edinburgh Mental Well-being Scale" ) OR KW ( WEMWBS OR SWEMWBS OR "Warwick-Edinburgh Mental Well-being Scale" )  27  S10 S6 OR S7 OR S8  4,372  S11 TI ( adult* or middle-age* OR "middle age*" OR working-age* OR "old people" OR elderly or "young adult*" ) OR AB ( adult* or middle-age* OR "middle age*" OR working-age* OR "old people" OR elderly or "young adult*" ) OR SU ( adult* or middle-age* OR "middle age*" OR working-age* OR "old people" OR elderly or "young adult*" ) OR KW ( adult* or middle-age* OR "middle age*" OR working-age* OR "old people" OR elderly or "young adult*" ) OR KA ( adult* or middle-age* OR "middle age*" OR working-age* OR "old people" OR elderly or "young adult*" ) OR TX ( adult* or middle-age* OR "middle age*" OR working-age* OR "old people" OR elderly or "young adult*" )  222,177  S12 S5 AND S10 AND S11  78 |
| Study records: |  |  |
| Data management | 11a | Search results are imported into RefWorks, reference management software (duplicates removed in Refworks). Titles and abstracts are imported into Refworks so they can be extracted for the screening process. Full articles are stored in the organisation’s secure database environments that are access controlled and backed up regularly. Access to the organisation’s servers is provided only for ICT maintenance purposes and to dedicated personnel. |
| Selection process | 11b | Two researchers independently carry out the screening process. First, titles and abstracts are screened for relevance and eligibility. Articles not relevant or not meeting the inclusion criteria are removed. Articles with insufficient information to determine relevance are screened in phase 2. In phase 2, full texts of the remaining articles are screened against the inclusion criteria. Disagreements are discussed until consensus is agreed. |
| Data collection process | 11c | Data extraction form is developed to include information on population, methodology, and outcomes. Data extraction is carried out by the principal reviewer, other research members verifying the data. |
| Data items | 12 | Data extracted: study identification features: authors, title, country, year; study characteristics: aims/objectives, study design, data source, data collection method; population characteristics, sample size; PMH measures, PMH outcome results/key findings; and study limitations/ strengths.  Each included article is analysed and data extracted with the aid of Atlas.ti (data analysis software). |
| Outcomes and prioritization | 13 | Our target population is those living alone; one-person households.  Primary outcome is (experienced) positive mental health (PMH) or mental wellbeing understood as the same as PMH using WEMWBS/SWEMWBS or WHO-5 measurements as both instruments include and measure both eudaimonic and hedonic elements of positive mental health/mental wellbeing. |
| Risk of bias in individual studies | 14 | Quality of reporting assessment using a modified checklist based on the Strengthening the Reporting of Observational Studies in Epidemiology (STROBE) guidelines and a checklist of good practice in the reporting of survey research. Two researchers from the team independently assess the studies. Discrepancies are discussed and resolved through consensus.  Updated March 2019 (addition): new, more appropriate quality assessment tool utilised: JBI Critical Appraisal Checklist for Prevalence Studies which covers: sample frame appropriateness, recruitment appropriateness, sample size, description of subjects and setting, coverage of data analysis, ascertainment and measurement of the condition, thoroughness of reporting statistical analysis, and adequacy and management of response rate.  Two researchers independently assess the studies. Discrepancies are discussed and resolved through consensus. |
| Data synthesis | 15d | A systematic narrative synthesis is provided with information presented in the text and tables to summarise and explain the characteristics and findings of the included studies. The narrative synthesis explores the relationship and findings both within and between the included studies. |
