# Supplementary material for: Living alone and positive mental health: a systematic review
Source: Syst Rev. 2019 Jun 7;8:134. doi: 10.1186/s13643-019-1057-x (PMC6555743; doi:10.1186/s13643-019-1057-x)
Supplement: Supplementary file 3 — Search strategies by database. This file presents the search strategies by each database employed in the study. (DOCX 35 kb) [file 13643_2019_1057_MOESM3_ESM.docx]

**Living alone and positive mental health: a systematic review**

## Literature search: 18.10.2017-9.11.2017

## Search strategies by database

**Ovid MEDLINE(R) Epub Ahead of Print, In-Process & Other Non-Indexed Citations, Ovid MEDLINE(R) Daily and Ovid MEDLINE(R) <1946 to Present>**18.10.2017

--------------------------------------------------------------------------------

1 "Living alone".mp. (3301)

2 ((living or life or live) adj3 single).ti,ab,kw. (3917)

3 ((living or life or live) adj3 alone).ti,ab,kw. (4248)

4 Single Person/ (2391)

5 (singled or "the single").ti,ab,kw. (1459848)

6 (single adj3 person*).ti,ab,kw. (1255)

7 (single adj3 people).ti,ab,kw. (391)

8 (single adj3 (women or men or adult*)).ti,ab,kw. (6031)

9 (("one person" or one-person or single) adj3 household*).ti,ab,kw. (758)

10 1 or 2 or 4 or 6 or 7 or 9 (11372)

11 Positive mental health.mp. (489)

12 ("mental well-being" or "mental well being" or "mental wellbeing").ti,ab,kw. (2098)

13 (WEMWBS or SWEMWBS or "Warwick-Edinburgh Mental Well-being Scale").ti,ab,kw. (129)

14 ("subjective well-being" or "subjective well being" or "subjective wellbeing").ti,ab,kw. (2724)

15 (WHO-5 or "WHO Five" or "WHO-5 Well-being Index").ti,ab,kw. (393)

16 11 or 12 or 13 or 14 or 15 (5610)

17 10 and 16 (36)

***************************

**Web of Science 25.9.2017**

(TS=("Living alone") OR TS=((living OR life OR live) NEAR/2 single*) OR TS=((living OR life OR live OR lived) NEAR/2 alone) OR TS=("one person" NEAR/2 household*) OR TS=(single* NEAR/2 (household* OR people OR person*)) OR TS= ("singlehood") OR TS=("single" NEAR/2 ("men" OR "women" OR "adult*" OR "middle-age*" OR working$age* OR "old* people" OR "elderly" ))) AND TS=("Positive mental health" OR "Mental wellbeing" OR "Mental well-being" OR "mental well being" OR "WEMWBS" OR "SWEMWBS" OR "Warwick-Edinburgh Mental Well-being Scale" OR “subjective well-being” OR “subjective wellbeing” OR “subjective well being”OR “WHO-5” OR “WHO-Five” OR “WHO-5 Well-being Index”)

Timespan: 1975-2017. Indexes: SCI-EXPANDED, SSCI, A&HCI, ESCI.

*[67 results]*

Science Citation Index Expanded (SCI-EXPANDED) --1975-present

Social Sciences Citation Index (SSCI) --1975-present

Arts & Humanities Citation Index (A&HCI) --1975-present

Emerging Sources Citation Index (ESCI) --2015-present

**ASSIA (ProQuest) 18.10.2017**

(((SU.EXACT("Single persons")) OR (ti((single NEAR/2 people) OR (single NEAR/2 person*) OR (single-living) OR ("singlehood" OR Bachelor OR bachelors OR bachelorhood OR Spinster OR Spinsters OR spinsterhood OR Unmarried)) OR ab((single NEAR/2 people) OR (single NEAR/2 person*) OR (single-living) OR (singlehood OR bachelor OR bachelors OR bachelorhood OR Spinster OR Spinsters OR spinsterhood OR Unmarried))) OR (ti((live OR life OR living OR lived) NEAR/2 alone) OR ab((live OR life OR living OR lived) NEAR/2 alone)) OR (ti((live OR life OR living OR lived) NEAR/2 single) OR ab((live OR life OR living OR lived) NEAR/2 single)) OR (ti(("one person" OR one-person) NEAR/2 household*) OR ab(("one person" OR one-person) NEAR/2 household*)) OR (ti(single NEAR/2 household*) OR ab(single NEAR/2 household*)) OR (ti((single NEAR/2 (men OR women OR adult* OR elderly OR old* OR student*))) OR ab((single NEAR/2 (men OR women OR adult* OR elderly OR old* OR student*))))) AND (ti("positive mental health" OR "mental well-being" OR "mental wellbeing" OR "mental well being" OR WEMWBS OR SWEMWBS OR "Warwick-Edinburgh Mental Well-being Scale") OR ab("positive mental health" OR "mental well-being" OR "mental wellbeing" OR "mental well being" OR WEMWBS OR SWEMWBS OR "Warwick-Edinburgh Mental Well-being Scale" OR “subjective well-being” OR “subjective wellbeing” OR “subjective well being” OR WHO-5 OR WHO-Five OR “WHO-5 Well-being Index”)))

**IBSS (ProQuest) 18.10.2017**

((SU.EXACT("Single persons")) OR (ti((single NEAR/2 people) OR (single NEAR/2 person*) OR (single-living) OR (“singlehood”)) OR ab((single NEAR/2 people) OR (single NEAR/2 person*) OR (single-living) OR (inglewood))) OR (ti((live OR life OR living OR lived) NEAR/2 alone) OR ab((live OR life OR living OR lived) NEAR/2 alone)) OR (ti((live OR life OR living OR lived) NEAR/2 single) OR ab((live OR life OR living OR lived) NEAR/2 single)) OR (ti(("one person" OR one-person) NEAR/4 household*) OR ab(("one person" OR one-person) NEAR/4 household*)) OR (ti(single NEAR/2 household*) OR ab(single NEAR/2 household*)) OR (ti((single NEAR/2 (men OR women OR adult* OR elderly OR old*))) OR ab((single NEAR/2 (men OR women OR adult* OR elderly OR old*))))) AND (ti("positive mental health" OR "mental well-being" OR "mental wellbeing" OR "mental well being" OR WEMWBS OR SWEMWBS OR "Warwick-Edinburgh Mental Well-being Scale" OR “subjective well-being” OR “subjective wellbeing” OR “subjective well being” OR “WHO-5” OR “WHO-Five” OR “WHO-5 Well-being Index”) OR ab("positive mental health" OR "mental well-being" OR "mental wellbeing" OR "mental well being" OR WEMWBS OR SWEMWBS OR "Warwick-Edinburgh Mental Well-being Scale" OR “subjective well-being” OR “subjective wellbeing” OR “subjective well being” OR “WHO-5” OR “WHO-Five” OR “WHO-5 Well-being Index”)) *[ 14 results]*

**Political Science Database (1985 -current) (ProQuest) 18.10.2017**Search strategy see: search strategy in IBSS
*[ 3 results]*

**Social Science Database (PQ) 18.10.2017**Search strategy see: search strategy in IBSS
*[ 27 results]*

**Sociology Database 18.10.2017***Search strategy see: search strategy in IBSS
[ 24 results]*

**Education database (1988-current) PQ) 18.10.2017**
Search strategy see: search strategy in IBSS
*[ 8 results]*

**Sociological Abstracts (ProQuest) 18.10.2017**

((SU.EXACT("Single persons")) OR (ti((single NEAR/2 people) OR (single NEAR/2 person*) OR (single-living) OR (singlehood OR Bachelor OR bachelors OR bachelorhood OR Spinster OR Spinsters OR spinsterhood OR Unmarried)) OR ab((single NEAR/2 people) OR (single NEAR/2 person*) OR (single-living) OR (singlehood OR bachelor OR bachelors OR bachelorhood OR Spinster OR Spinsters OR spinsterhood OR Unmarried ))) OR (ti((live OR life OR living OR lived) NEAR/2 alone) OR ab((live OR life OR living OR lived) NEAR/2 alone)) OR (ti((live OR life OR living OR lived) NEAR/2 single) OR ab((live OR life OR living OR lived) NEAR/2 single)) OR (ti(("one person" OR one-person) NEAR/2 household*) OR ab(("one person" OR one-person) NEAR/2 household*)) OR (ti(single NEAR/2 household*) OR ab(single NEAR/2 household*)) OR (ti((single NEAR/2 (men OR women OR adult* OR elderly OR old* OR student*))) OR ab((single NEAR/2 (men OR women OR adult* OR elderly OR old* OR student*))))) AND (ti("positive mental health" OR "mental well-being" OR "mental wellbeing" OR "mental well being" OR WEMWBS OR SWEMWBS OR "Warwick-Edinburgh Mental Well-being Scale" OR “subjective well-being” OR “subjective wellbeing” OR “subjective well being” OR “WHO-5” OR “WHO-Five” OR “WHO-5 Well-being Index”) OR ab("positive mental health" OR "mental well-being" OR "mental wellbeing" OR "mental well being" OR WEMWBS OR SWEMWBS OR "Warwick-Edinburgh Mental Well-being Scale" OR “subjective well-being” OR “subjective wellbeing” OR “subjective well being” OR “WHO-5” OR “WHO-Five” OR “WHO-5 Well-being Index”))

*[49 results]*

**Cochrane Library (Wiley) 18.10.2017**

a)

There is 1 result from 1087263 records for your search on '"living alone" OR "single persons" OR "single person" OR "single people" OR single-living OR "one-person household" OR "one person household" OR singlehood OR "single men" OR "single women" OR "single elderly" OR "single old" OR "single adult" OR "single adults" in Title, Abstract, Keywords and "positive mental health" OR "mental wellbeing" OR "mental well being" OR "mental well-being" OR "subjective well-being" OR "subjective wellbeing" OR "subjective well being" OR "WHO-5" OR "WHO-Five" OR "WHO-5 Well-being Index" in Title, Abstract, Keywords in Trials'
Cochrane Central Register of Controlled Trials : Issue 9 of 12, September 2017

b)
There is 1 result from 9994 records for your search on '"living alone" OR "single persons" OR "single person" OR "single people" OR single-living OR "one-person household" OR "one person household" in Tables and "positive mental health" OR "mental wellbeing" OR "mental well being" OR "mental well-being" OR "subjective well-being" OR "subjective wellbeing" OR "subjective well being" OR "WHO-5" OR "WHO-Five" OR "WHO-5 Well-being Index" in Tables in Cochrane Reviews'
Cochrane Database of Systematic Reviews : Issue 10 of 12, October 2017

**CINAHL (EbscoHost) 24.10.2017**

#Query
S1 (MH "Single Person+") OR (MH "Single Women") OR (MH "Single Men")

S2 TI ( (live OR life OR living) W2 alone ) OR AB ( (live OR life OR living) W2 alone ) OR SU ( (live OR life OR living) W2 alone )

S3 TI ( (live OR life OR living) W2 single ) OR AB ( (live OR life OR living) W2 single ) OR SU ( (live OR life OR living) W2 single )

S4 TI ( "single-living" OR singlehood ) OR AB ( "single-living" OR singlehood ) OR SU ( "single-living" OR singlehood )

S5 TI ("one-person household*" OR "one person household*") OR AB("one-person household*" OR "one person household*) OR SU ("one-person household*" OR "one person household*")

S6 TI ("single-person household*" OR "single person household*") OR AB("single-person household*" OR "single person household*") OR SU("single-person household*" OR "single person household*")

S7 TI ( "single person*" OR "single people" OR "single men" OR "single women" ) OR AB ( "single person*" OR "single people" OR "single men" OR "single women" ) OR SU ( "single person*" OR "single people" OR "single men" OR "single women" )

S8 S1 OR S2 OR S3 OR S4 OR S5 OR S6 OR S7

S9 TI positive W1 "mental health" OR AB positive W1 "mental health" OR SU positive W1 "mental health"

S10 TI ( mental W1 (well-being OR wellbeing OR "well being") ) OR AB ( mental W1 (well-being OR wellbeing OR "well being") ) OR SU ( mental W1 (well-being OR wellbeing OR "well being") )

S11 TI ( WEMWBS or SWEMWBS or "Warwick-Edinburgh Mental Well-being Scale" ) OR AB ( WEMWBS or SWEMWBS or "Warwick-Edinburgh Mental Well-being Scale" ) OR SU ( WEMWBS or SWEMWBS or "Warwick-Edinburgh Mental Well-being Scale" )

S12 TI (“subjective well-being” OR “subjective wellbeing” OR “subjective well being” OR WHO-5 OR WHO-Five OR “WHO-5 Well-being Index”) OR AB (“subjective well-being” OR “subjective wellbeing” OR “subjective well being” OR WHO-5 OR WHO-Five OR “WHO-5 Well-being Index”) OR SU (“subjective well-being” OR “subjective wellbeing” OR “subjective well being” OR WHO-5 OR WHO-Five OR “WHO-5 Well-being Index”)

S13 S9 OR S10 OR S11 OR S12

S14 S8 AND S13 13
Search modes - Boolean/Phrase Interface - EBSCOhost Research Databases Search Screen - Advanced Search Database – CINAHL

**ASE Academic Search Elite (EbscoHost) 25.10.2017**

| S1 | (DE "SINGLE people" OR DE "LIVING alone" OR DE "SINGLE men" OR DE "SINGLE women") |  | Interface - EBSCOhost Research Databases  Search Screen - Advanced Search  Database - Academic Search Elite | 2,422 |
| --- | --- | --- | --- | --- |
| S2 | TI ( (live OR life OR living) W2 alone ) OR AB ( (live OR life OR living) W2 alone ) OR KW ( (live OR life OR living) W2 alone ) |  | Interface - EBSCOhost Research Databases  Search Screen - Basic Search  Database - Academic Search Elite | 2,721 |
| S3 | TI ( (live OR life OR living) W1 "by oneself" ) OR AB ( (live OR life OR living) W1 "by oneself" ) OR KW ( (live OR life OR living) W1 "by oneself") |  | Interface - EBSCOhost Research Databases  Search Screen - Advanced Search  Database - Academic Search Elite | 49 |
| S4 | TI ( (live OR life OR living) W2 single ) OR AB ( (live OR life OR living) W2 single ) OR KW ( (live OR life OR living) W2 single ) |  | Interface - EBSCOhost Research Databases  Search Screen - Basic Search  Database - Academic Search Elite | 1,477 |
| S5 | TI ( "single-living" OR singlehood ) OR AB ( "single-living" OR singlehood ) OR KW ( "single-living" OR singlehood ) |  | Interface - EBSCOhost Research Databases  Search Screen - Basic Search  Database - Academic Search Elite | 631 |
| S6 | TI ("one-person household*" OR "one person household*") OR AB("one-person household*" OR "one person household*) OR KW("one-person household*" OR "one person household*") |  | Interface - EBSCOhost Research Databases  Search Screen - Basic Search  Database - Academic Search Elite | 41 |
| S7 | TI ("single-person household*" OR "single person household*") OR AB("single-person household*" OR "single person household*") OR KW("single-person household*" OR "single person household*") |  | Interface - EBSCOhost Research Databases  Search Screen - Basic Search  Database - Academic Search Elite | 95 |
| S8 | TI ( "single person*" OR "single people" OR "single men" OR "single women" ) OR AB ( "single person*" OR "single people" OR "single men" OR "single women" ) OR KW ( "single person*" OR "single people" OR "single men" OR "single women" ) |  | Interface - EBSCOhost Research Databases  Search Screen - Basic Search  Database - Academic Search Elite | 2,765 |
| S9 | S1 OR S2 OR S3 OR S4 OR S5 OR S6 OR S7 OR S8 |  | Interface - EBSCOhost Research Databases  Search Screen - Basic Search  Database - Academic Search Elite | 8,873 |
| S10 | TI (positive W1 "mental health") OR AB (positive W1 "mental health") OR KW (positive W1 "mental health") |  | Interface - EBSCOhost Research Databases  Search Screen - Basic Search  Database - Academic Search Elite | 436 |
| S11 | TI ( mental W1 (well-being OR wellbeing OR "well being") ) OR AB ( mental W1 (well-being OR wellbeing OR "well being") ) OR KW ( mental W1 (well-being OR wellbeing OR "well being") ) |  | Interface - EBSCOhost Research Databases  Search Screen - Basic Search  Database - Academic Search Elite | 1,800 |
| S12 | TI ( WEMWBS or SWEMWBS or "Warwick-Edinburgh Mental Well-being Scale" ) OR AB ( WEMWBS or SWEMWBS or "Warwick-Edinburgh Mental Well-being Scale" ) OR KW ( WEMWBS or SWEMWBS or "Warwick-Edinburgh Mental Well-being Scale" ) |  | Interface - EBSCOhost Research Databases  Search Screen - Basic Search  Database - Academic Search Elite | 92 |
| S13 | DE "SUBJECTIVE well-being (Psychology)" OR TI (“subjective well-being” OR “subjective wellbeing” OR “subjective well being” OR WHO-5 OR WHO-Five OR “WHO-5 Well-being Index”) OR AB (“subjective well-being” OR “subjective wellbeing” OR “subjective well being” OR WHO-5 OR WHO-Five OR “WHO-5 Well-being Index”) OR KW (“subjective well-being” OR “subjective wellbeing” OR “subjective well being” OR WHO-5 OR WHO-Five OR “WHO-5 Well-being Index”) |  | Interface - EBSCOhost Research Databases  Search Screen - Basic Search  Database - Academic Search Elite | 2,208 |
| S14 | S10 OR S11 OR S12 OR S13 |  | Interface - EBSCOhost Research Databases  Search Screen - Basic Search  Database - Academic Search Elite | 6 |

**S15** S 9 AND S14 41

**SOCINDXFullText (EbscoHost) 25.10.2017**

Search strategy: same as in Academic Search Elite. Results 25

**AgeLine (EbscoHost) 26.10.017**

| S1 | (DE "LIVING alone") |  | Interface - EBSCOhost Research Databases  Search Screen - Advanced Search  Database - AgeLine | 412 |
| --- | --- | --- | --- | --- |
| S2 | TI ( (live OR life OR living) W2 alone ) OR AB ( (live OR life OR living) W2 alone ) OR SU ( (live OR life OR living) W2 alone ) |  | Interface - EBSCOhost Research Databases  Search Screen - Advanced Search  Database - AgeLine | 1,611 |
| S3 | TI ( (live OR life OR living) W1 "by oneself" ) OR AB ( (live OR life OR living) W1 "by oneself" ) OR SU ( (live OR life OR living) W1 "by oneself") |  | Interface - EBSCOhost Research Databases  Search Screen - Advanced Search  Database - AgeLine | 6 |
| S4 | TI ( (live OR life OR living) W2 single ) OR AB ( (live OR life OR living) W2 single ) OR SU ( (live OR life OR living) W2 single ) |  | Interface - EBSCOhost Research Databases  Search Screen - Advanced Search  Database - AgeLine | 80 |
| S5 | TI ( "single-living" OR singlehood ) OR AB ( "single-living" OR singlehood ) OR SU ( "single-living" OR singlehood ) |  | Interface - EBSCOhost Research Databases  Search Screen - Advanced Search  Database - AgeLine | 47 |
| S6 | TI ("one-person household*" OR "one person household*") OR AB("one-person household*" OR "one person household*) OR SU ("one-person household*" OR "one person household*") |  | Interface - EBSCOhost Research Databases  Search Screen - Advanced Search  Database - AgeLine | 10 |
| S7 | TI ("single-person household*" OR "single person household*") OR AB("single-person household*" OR "single person household*") OR SU ("single-person household*" OR "single person household*") |  | Interface - EBSCOhost Research Databases  Search Screen - Advanced Search  Database - AgeLine | 28 |
| S8 | TI ( "single person*" OR "single people" OR "single men" OR "single women" ) OR AB ( "single person*" OR "single people" OR "single men" OR "single women" ) OR SU ( "single person*" OR "single people" OR "single men" OR "single women" ) |  | Interface - EBSCOhost Research Databases  Search Screen - Advanced Search  Database - AgeLine | 317 |
| S9 | S1 OR S2 OR S3 OR S4 OR S5 OR S6 OR S7 OR S8 |  | Interface - EBSCOhost Research Databases  Search Screen - Advanced Search  Database - AgeLine | 2,007 |
| S10 | TI (positive W1 "mental health") OR AB (positive W1 "mental health") OR SU (positive W1 "mental health") |  | Interface - EBSCOhost Research Databases  Search Screen - Advanced Search  Database - AgeLine | 35 |
| S11 | TI ( mental W1 (well-being OR wellbeing OR "well being") ) OR AB ( mental W1 (well-being OR wellbeing OR "well being") ) OR SU ( mental W1 (well-being OR wellbeing OR "well being") ) |  | Interface - EBSCOhost Research Databases  Search Screen - Advanced Search  Database - AgeLine | 149 |
| S12 | TI ( WEMWBS or SWEMWBS or "Warwick-Edinburgh Mental Well-being Scale" ) OR AB ( WEMWBS or SWEMWBS or "Warwick-Edinburgh Mental Well-being Scale" ) OR SU ( WEMWBS or SWEMWBS or "Warwick-Edinburgh Mental Well-being Scale" ) |  | Interface - EBSCOhost Research Databases  Search Screen - Advanced Search  Database - AgeLine | 2 |
| S13 | TI (“subjective well-being” OR “subjective wellbeing” OR “subjective well being” OR WHO-5 OR WHO-Five OR “WHO-5 Well-being Index”) OR AB (“subjective well-being” OR “subjective wellbeing” OR “subjective well being” OR WHO-5 OR WHO-Five OR “WHO-5 Well-being Index”) OR SU (“subjective well-being” OR “subjective wellbeing” OR “subjective well being” OR WHO-5 OR WHO-Five OR “WHO-5 Well-being Index”) |  | Interface - EBSCOhost Research Databases  Search Screen - Advanced Search  Database - AgeLine | 182 |
| S14 | S10 OR S11 OR S12 OR S13 |  | Interface - EBSCOhost Research Databases  Search Screen - Advanced Search  Database - AgeLine | 6 |

S15 S9 AND S14 19

**PsycINFO (EbscoHost) 26.10.2017**

S1 (DE "LIVING alone" OR DE "Single Persons")

S2 TI ( (live OR life OR living) W2 alone ) OR AB ( (live OR life OR living) W2 alone ) OR SU ( (live OR life OR living) W2 alone ) OR KW ( (live OR life OR living) W2 alone )

S3 TI ( (live OR life OR living) W1 "by oneself" ) OR AB ( (live OR life OR living) W1 "by oneself" ) OR SU ( (live OR life OR living) W1 "by oneself") KW ( (live OR life OR living) W1 "by oneself")

S4 TI ( (live OR life OR living) W2 single ) OR AB ( (live OR life OR living) W2 single ) OR SU ( (live OR life OR living) W2 single ) OR KW ( (live OR life OR living) W2 single )

S5 TI ( "single-living" OR singlehood ) OR AB ( "single-living" OR singlehood ) OR SU ( "single-living" OR singlehood ) OR KW ( "single-living" OR singlehood )

S6 TI ("one-person household*" OR "one person household*") OR AB("one-person household*" OR "one person household*) OR SU ("one-person household*" OR "one person household*") KW ("one-person household*" OR "one person household*")

S7 TI ("single-person household*" OR "single person household*") OR AB("single-person household*" OR "single person household*") OR SU ("single-person household*" OR "single person household*") OR KW ("single-person household*" OR "single person household*")

S8 TI ( "single person*" OR "single people" OR "single men" OR "single women" ) OR AB ( "single person*" OR "single people" OR "single men" OR "single women" ) OR SU ( "single person*" OR "single people" OR "single men" OR "single women" ) OR KW ( "single person*" OR "single people" OR "single men" OR "single women" )

S9 S1 OR S2 OR S3 OR S4 OR S5 OR S6 OR S7 OR S8

S10 TI (positive W1 "mental health") OR AB (positive W1 "mental health") OR SU (positive W1 "mental health") OR KW (positive W1 "mental health")

S11 TI ( mental W1 (well-being OR wellbeing OR "well being") ) OR AB ( mental W1 (well-being OR wellbeing OR "well being") ) OR SU ( mental W1 (well-being OR wellbeing OR "well being") ) OR KW ( mental W1 (well-being OR wellbeing OR "well being") )

S12 TI ( WEMWBS or SWEMWBS or "Warwick-Edinburgh Mental Well-being Scale" ) OR AB ( WEMWBS or SWEMWBS or "Warwick-Edinburgh Mental Well-being Scale" ) OR SU ( WEMWBS or SWEMWBS or "Warwick-Edinburgh Mental Well-being Scale" ) OR KW ( WEMWBS or SWEMWBS or "Warwick-Edinburgh Mental Well-being Scale" )

S13 TI (“subjective well-being” OR “subjective wellbeing” OR “subjective well being” OR WHO-5 OR WHO-Five OR “WHO-5 Well-being Index”) OR AB (“subjective well-being” OR “subjective wellbeing” OR “subjective well being” OR WHO-5 OR WHO-Five OR “WHO-5 Well-being Index”) OR SU (“subjective well-being” OR “subjective wellbeing” OR “subjective well being” OR WHO-5 OR WHO-Five OR “WHO-5 Well-being Index”)OR KW (“subjective well-being” OR “subjective wellbeing” OR “subjective well being” OR WHO-5 OR WHO-Five OR “WHO-5 Well-being Index”)

S14 S10 OR S11 OR S12 OR S12 OR S13

S15 S9 AND S14 48

**Urban Studies Abstracts (EbscoHost) 28.9.2017**
S1 (DE "LIVING alone" OR DE "Single Persons")

S2 TI ( (live OR life OR living) W2 alone ) OR AB ( (live OR life OR living) W2 alone ) OR SU ( (live OR life OR living) W2 alone ) OR KW ( (live OR life OR living) W2 alone )

S3 TI ( (live OR life OR living) W1 "by oneself" ) OR AB ( (live OR life OR living) W1 "by oneself" ) OR SU ( (live OR life OR living) W1 "by oneself") KW ( (live OR life OR living) W1 "by onesel

S4 TI ( (live OR life OR living) W2 single ) OR AB ( (live OR life OR living) W2 single ) OR SU ( (live OR life OR living) W2 single ) OR KW ( (live OR life OR living) W2 single )

S5 TI ( "single-living" OR singlehood ) OR AB ( "single-living" OR singlehood ) OR SU ( "single-living" OR singlehood ) OR KW ( "single-living" OR singlehood )

S6 TI ("one-person household*" OR "one person household*") OR AB("one-person household*" OR "one person household*) OR SU ("one-person household*" OR "one person household*") KW ("one-person household*" OR "one person household*")

S7 TI ("single-person household*" OR "single person household*") OR AB("single-person household*" OR "single person household*") OR SU ("single-person household*" OR "single person household*") OR KW ("single-person household*" OR "single person household*")

S8 TI ( "single person*" OR "single people" OR "single men" OR "single women" ) OR AB ( "single person*" OR "single people" OR "single men" OR "single women" ) OR SU ( "single person*" OR "single people" OR "single men" OR "single women" ) OR KW ( "single person*" OR "single people" OR "single men" OR "single women" )

S9 S1 OR S2 OR S3 OR S4 OR S5 OR S6 OR S7 OR S8

S10 TI ( WEMWBS or SWEMWBS or "Warwick-Edinburgh Mental Well-being Scale" ) OR AB ( WEMWBS or SWEMWBS or "Warwick-Edinburgh Mental Well-being Scale" ) OR SU ( WEMWBS or SWEMWBS or "Warwick-Edinburgh Mental Well-being Scale" ) OR KW ( WEMWBS or SWEMWBS or "Warwick-Edinburgh Mental Well-being Scale" )

S11 TI ( health OR well-being OR "well being" OR wellbeing ) OR AB ( health OR well-being OR "well being" OR wellbeing ) OR KW ( health OR well-being OR "well being" OR wellbeing ) OR SU ( health OR well-being OR "well being" OR wellbeing )

S12 TI (“subjective well-being” OR “subjective wellbeing” OR “subjective well being” OR WHO-5 OR WHO-Five OR “WHO-5 Well-being Index”) OR AB (“subjective well-being” OR “subjective wellbeing” OR “subjective well being” OR WHO-5 OR WHO-Five OR “WHO-5 Well-being Index”) OR KW (“subjective well-being” OR “subjective wellbeing” OR “subjective well being” OR WHO-5 OR WHO-Five OR “WHO-5 Well-being Index”) OR SU (“subjective well-being” OR “subjective wellbeing” OR “subjective well being” OR WHO-5 OR WHO-Five OR “WHO-5 Well-being Index”)

S13 S10 OR S11 OR S12

S14 S9 AND S13 16

**Google Scholar**

intitle:"positive mental health" OR intitle:"mental well being" OR intitle:"subjective well being" OR intitle:WHO-5 OR intitle:WHO-five OR intitle:"Warwick-Edinburgh Mental Well-being Scale"
"living alone" OR "single living" OR "one person household"
280

**Updated search 2019**

**Database: Ovid MEDLINE(R) and Epub Ahead of Print, In-Process & Other Non-Indexed Citations and Daily <1946 to May 03, 2019>**

**Search Strategy: May 9^th^, 2019**

--------------------------------------------------------------------------------

1 "Living alone".mp. (3469)

2 ((living or life or live) adj3 single).ti,ab,kw. (4084)

3 ((living or life or live) adj3 alone).ti,ab,kw. (4428)

4 Single Person/ (2290)

5 (singled or "the single").ti,ab,kw. (1495607)

6 (single adj3 person*).ti,ab,kw. (1293)

7 (single adj3 people).ti,ab,kw. (414)

8 (single adj3 (women or men or adult*)).ti,ab,kw. (6127)

9 (("one person" or one-person or single) adj3 household*).ti,ab,kw. (780)

10 1 or 2 or 4 or 6 or 7 or 9 (11702)

11 Positive mental health.mp. (575)

12 ("mental well-being" or "mental well being" or "mental wellbeing").ti,ab,kw. (2441)

13 (WEMWBS or SWEMWBS or "Warwick-Edinburgh Mental Well-being Scale").ti,ab,kw. (151)

14 ("subjective well-being" or "subjective well being" or "subjective wellbeing").ti,ab,kw. (3040)

15 (WHO-5 or "WHO Five" or "WHO-5 Well-being Index").ti,ab,kw. (463)

16 11 or 12 or 13 or 14 or 15 (6401)

17 10 and 16 (47)

18 limit 17 to yr="2017 -Current" (16)

***************************

**Web of Science May, 9^th^ 2019**

(TS=("Living alone") OR TS=((living OR life OR live) NEAR/2 single*) OR TS=((living OR life OR live OR lived) NEAR/2 alone) OR TS=("one person" NEAR/2 household*) OR TS=(single* NEAR/2 (household* OR people OR person*)) OR TS= ("singlehood") OR TS=("single" NEAR/2 ("men" OR "women" OR "adult*" OR "middle-age*" OR working$age* OR "old* people" OR "elderly" ))) AND TS=("Positive mental health" OR "Mental wellbeing" OR "Mental well-being" OR "mental well being" OR "WEMWBS" OR "SWEMWBS" OR "Warwick-Edinburgh Mental Well-being Scale" OR "subjective well-being" OR "subjective wellbeing" OR "subjective well being"OR "WHO-5" OR "WHO-Five" OR "WHO-5 Well-being Index")

Timespan: 2017-2019. Indexes: SCI-EXPANDED, SSCI, A&HCI, ESCI.
Results 19

**Cochrane Library (Wiley)**

a) Cochrane Central Register of Controlled Trials

2 Trials matching on "living alone" OR "single persons" OR "single person" OR "single people" OR single-living OR "one-person household" OR "one person household" in Title Abstract Keyword AND "positive mental health" OR "mental wellbeing" OR "mental well being" OR "mental well-being" OR "subjective well-being" OR "subjective wellbeing" OR "subjective well being" OR "WHO-5" OR "WHO-Five" OR "WHO-5 Well-being Index" in Title Abstract Keyword - with Cochrane Library publication date Between Sep 2017 and May 2019 (Word variations have been searched)

Cochrane Central Register of Controlled Trials

Issue 5 of 12, May 2019

b) Cochrane Database of Systematic Reviews

0 Cochrane Reviews matching on "living alone" OR "single persons" OR "single person" OR "single people" OR single-living OR "one-person household" OR "one person household" in Title Abstract Keyword AND "positive mental health" OR "mental wellbeing" OR "mental well being" OR "mental well-being" OR "subjective well-being" OR "subjective wellbeing" OR "subjective well being" OR "WHO-5" OR "WHO-Five" OR "WHO-5 Well-being Index" in Title Abstract Keyword - with Cochrane Library publication date Between Sep 2017 and May 2019 (Word variations have been searched)

Cochrane Database of Systematic Reviews

Issue 5 of 12, May 2019

**CINAHL (EbscoHost)**

S1 (MH "Single Person+") OR (MH "Single Women") OR (MH "Single Men")

S2 TI ( (live OR life OR living) W2 alone ) OR AB ( (live OR life OR living) W2 alone ) OR SU ( (live OR life OR living) W2 alone )

S3 TI ( (live OR life OR living) W2 single ) OR AB ( (live OR life OR living) W2 single ) OR SU ( (live OR life OR living) W2 single )

S4 TI ( "single-living" OR singlehood ) OR AB ( "single-living" OR singlehood ) OR SU ( "single-living" OR singlehood )

S5 TI ("one-person household*" OR "one person household*") OR AB("one-person household*" OR "one person household*) OR SU ("one-person household*" OR "one person household*")

S6 TI ("single-person household*" OR "single person household*") OR AB("single-person household*" OR "single person household*") OR SU("single-person household*" OR "single person household*")

S7 TI ( "single person*" OR "single people" OR "single men" OR "single women" ) OR AB ( "single person*" OR "single people" OR "single men" OR "single women" ) OR SU ( "single person*" OR "single people" OR "single men" OR "single women" )

S8 S1 OR S2 OR S3 OR S4 OR S5 OR S6 OR S7

S9 TI positive W1 "mental health" OR AB positive W1 "mental health" OR SU positive W1 "mental health"

S10 TI ( mental W1 (well-being OR wellbeing OR "well being") ) OR AB ( mental W1 (well-being OR wellbeing OR "well being") ) OR SU ( mental W1 (well-being OR wellbeing OR "well being") )

S11 TI ( WEMWBS or SWEMWBS or "Warwick-Edinburgh Mental Well-being Scale" ) OR AB ( WEMWBS or SWEMWBS or "Warwick-Edinburgh Mental Well-being Scale" ) OR SU ( WEMWBS or SWEMWBS or "Warwick-Edinburgh Mental Well-being Scale" )

S12 TI (“subjective well-being” OR “subjective wellbeing” OR “subjective well being” OR WHO-5 OR WHO-Five OR “WHO-5 Well-being Index”) OR AB (“subjective well-being” OR “subjective wellbeing” OR “subjective well being” OR WHO-5 OR WHO-Five OR “WHO-5 Well-being Index”) OR SU (“subjective well-being” OR “subjective wellbeing” OR “subjective well being” OR WHO-5 OR WHO-Five OR “WHO-5 Well-being Index”)

S13 S9 OR S10 OR S11 OR S12

S14 S8 AND S13
View Results (9)

Limiters - Published Date: 20170101-20191231

Search modes - Boolean/Phrase

**PsycINFO (EbscoHost)**

S1 (DE "LIVING alone" OR DE "Single Persons")

S2 TI ( (live OR life OR living) W2 alone ) OR AB ( (live OR life OR living) W2 alone ) OR SU ( (live OR life OR living) W2 alone ) OR KW ( (live OR life OR living) W2 alone )

S3 TI ( (live OR life OR living) W1 "by oneself" ) OR AB ( (live OR life OR living) W1 "by oneself" ) OR SU ( (live OR life OR living) W1 "by oneself") KW ( (live OR life OR living) W1 "by oneself")

S4 TI ( (live OR life OR living) W2 single ) OR AB ( (live OR life OR living) W2 single ) OR SU ( (live OR life OR living) W2 single ) OR KW ( (live OR life OR living) W2 single )

S5 TI ( "single-living" OR singlehood ) OR AB ( "single-living" OR singlehood ) OR SU ( "single-living" OR singlehood ) OR KW ( "single-living" OR singlehood )

S6 TI ("one-person household*" OR "one person household*") OR AB("one-person household*" OR "one person household*) OR SU ("one-person household*" OR "one person household*") KW ("one-person household*" OR "one person household*")

S7 TI ("single-person household*" OR "single person household*") OR AB("single-person household*" OR "single person household*") OR SU ("single-person household*" OR "single person household*") OR KW ("single-person household*" OR "single person household*")

S8 TI ( "single person*" OR "single people" OR "single men" OR "single women" ) OR AB ( "single person*" OR "single people" OR "single men" OR "single women" ) OR SU ( "single person*" OR "single people" OR "single men" OR "single women" ) OR KW ( "single person*" OR "single people" OR "single men" OR "single women" )

S9 S1 OR S2 OR S3 OR S4 OR S5 OR S6 OR S7 OR S8

S10 TI (positive W1 "mental health") OR AB (positive W1 "mental health") OR SU (positive W1 "mental health") OR KW (positive W1 "mental health")

S11 TI ( mental W1 (well-being OR wellbeing OR "well being") ) OR AB ( mental W1 (well-being OR wellbeing OR "well being") ) OR SU ( mental W1 (well-being OR wellbeing OR "well being") ) OR KW ( mental W1 (well-being OR wellbeing OR "well being") )

S12 TI ( WEMWBS or SWEMWBS or "Warwick-Edinburgh Mental Well-being Scale" ) OR AB ( WEMWBS or SWEMWBS or "Warwick-Edinburgh Mental Well-being Scale" ) OR SU ( WEMWBS or SWEMWBS or "Warwick-Edinburgh Mental Well-being Scale" ) OR KW ( WEMWBS or SWEMWBS or "Warwick-Edinburgh Mental Well-being Scale" )

S13 TI (“subjective well-being” OR “subjective wellbeing” OR “subjective well being” OR WHO-5 OR WHO-Five OR “WHO-5 Well-being Index”) OR AB (“subjective well-being” OR “subjective wellbeing” OR “subjective well being” OR WHO-5 OR WHO-Five OR “WHO-5 Well-being Index”) OR SU (“subjective well-being” OR “subjective wellbeing” OR “subjective well being” OR WHO-5 OR WHO-Five OR “WHO-5 Well-being Index”)OR KW (“subjective well-being” OR “subjective wellbeing” OR “subjective well being” OR WHO-5 OR WHO-Five OR “WHO-5 Well-being Index”)

S14 S10 OR S11 OR S12 OR S12 OR S13

S15 S9 AND S14
View Results (9)

Limiters - Publication Year: 2017-2019

Search modes - Boolean/Phrase
