# Supplementary material for: Living alone and positive mental health: a systematic review
Source: Syst Rev. 2019 Jun 7;8:134. doi: 10.1186/s13643-019-1057-x (PMC6555743; doi:10.1186/s13643-019-1057-x)
Supplement: Supplementary file 4 — JBI critical appraisal checklist for prevalence studies. This file presents the JBI critical appraisal checklist for studies reporting prevalence data. (DOCX 17 kb) [file 13643_2019_1057_MOESM4_ESM.docx]

**Additional file 4.**

**JBI Critical Appraisal Checklist for Studies Reporting Prevalence Data**

Reviewer Date

Author Year Record Number

|  | Yes | No | Unclear | Not applicable |
| --- | --- | --- | --- | --- |
| 1. Was the sample frame appropriate to address the target population? | □ | □ | □ | □ |
| 1. Were study participants sampled in an appropriate way? | □ | □ | □ | □ |
| 1. Was the sample size adequate? | □ | □ | □ | □ |
| 1. Were the study subjects and the setting described in detail? | □ | □ | □ | □ |
| 1. Was the data analysis conducted with sufficient coverage of the identified sample? | □ | □ | □ | □ |
| 1. Were valid methods used for the identification of the condition? | □ | □ | □ | □ |
| 1. Was the condition measured in a standard, reliable way for all participants? | □ | □ | □ | □ |
| 1. Was there appropriate statistical analysis? | □ | □ | □ | □ |
| 1. Was the response rate adequate, and if not, was the low response rate managed appropriately? | □ | □ | □ | □ |

Overall appraisal: Include □ Exclude □ Seek further info □

Comments (Including reason for exclusion)
